# Supplementary material for: Cost-effectiveness of sentinel screening of endemic diseases alongside malaria diagnosis: A case study in schistosomiasis
Source: PLoS Negl Trop Dis. 2024 Jul 29;18(7):e0012339. doi: 10.1371/journal.pntd.0012339 (PMC11309411; doi:10.1371/journal.pntd.0012339)
Supplement: S1 Table — (DOCX) [file pntd.0012339.s001.docx]

**S1 Table . Complete list of model parameters**

| **Parameters** | **Value** | **Reference/assumptions** |
| --- | --- | --- |
|  |  |  |
| **Threshold (Willingness to pay)** | 799 | GDP per capita Uganda 2019 [1] |
| Prevalence Malaria (prevalence of malaria within febrile presentations at clinics) | 0.38 | [2] |
| Schistosomiasis | 0.26 | [3] |
|  |  |  |
| **SENSITIVITY and SPECIFICITY** | | |
| **Malaria** |  |  |
| Sensitivity RDT prototype | 0.98 | University of Glasgow personal communication |
| Specificity RDT prototype | 0.95 | University of Glasgow personal communication |
| Sensitivity routinely used mRDT | 0.96 | [4] |
| Specificity routinely used mRDT | 0.87 | [4] |
| **Schistosomiasis** |  |  |
| Sensitivity | 0.95 | University of Glasgow personal communication |
| Specificity | 0.90 | University of Glasgow personal communication |
| Sensitivity comparator (urine test) | 0.83 | [5] |
| Specificity comparator (urine test) | 0.81 | [5] |
|  |  |  |
| **MODEL transition PROBABILITIES** | | |
| **Malaria** |  |  |
| Firstline treatment success | 0.95 | [6] |
| Probability of severe malaria after correct treatment | 0.10 | [7] |
| Probability of severe malaria if untreated | 0.10 | Assumption: same probability of severe if correct treatment |
| Probability of inpatient care if severe malaria | 0.48 | [7] |
| Probability of outpatient care if no complications | 0.48 | [7] |
| Probability of death if severe malaria and inpatient care | 0.10 | [7] |
| Probability of death if severe malaria and self-treated | 0.25 | [7] |
| **Non-malaria febrile illness (NMFI)** |  |  |
| Probability of inpatient care if severe NMFI | 0.48 | [7] |
| Probability of outpatient care if no complications | 0.48 | [7] |
| Probability of death if severe NMFI and inpatient care | 0.15 | [7] |
| Probability of death if severe NMFI and self treated | 0.25 | [6] |
| Probability of malaria treatment efficacy for other bacterial infections | 0.75 | [7] |
| **Schistosomiasis** |  |  |
| Probability of advance stage of disease among infected | 0.25 | [8] Prevalence of anaemia in individuals with schistosomiasis, value on Tanzania, region close to Lake Victoria. |
| WHO MDA coverage | 1.00 | Assumption |
| WHO MDA coverage scenario | 0.75 | [9] |
| **COST  all costs in US$ before 2019 were converted to Ugandan shillings, inflated[1], and then converted to US$ at IMF historical exchange rates[10]** | | |
| Health service at clinic arrival | $2.00 | Cost of outpatient visit [11] minus costs of RDT and related additional costs (gloves, cotton, sanitiser) [12] |
| Multiplex RDT prototype cost | $1.20 | University of Glasgow personal communication |
| mRDT | $0.90 | [12] |
| Miscellaneous costs RDT related (gloves, cotton etc.) | $0.10 | [12] |
| RDT comparator cost schistosomiasis | $0.90 | Assumption (equal to mRDT) |
| Outpatient staying/healthcare costs | $3.00 | [3] |
| **Malaria** |  |  |
| Malaria cost of drugs (first line treatment) | $1.57 |  |
| Malaria cost of drugs (severe malaria) | $0.00 | Included in inpatient or outpatient costs |
| Inpatient care | $43.60 | [11] Inpatient costs for of 4 days (average children length of hospitalisation for malaria) |
| Outpatient care | $3.00 | [11] |
| **Non-malaria febrile illness (NMFI)** |  |  |
| NMFI cost of drugs (first line treatment) | $0.35 | [4] |
| NMFI cost of drugs (severe NMFI) | $0.00 | Included in inpatient or outpatient costs |
| NMFI inpatient care | $72.7 | Assumption on different costs between NMFI and malaria inpatient in [13] based on malaria inpatient care cost |
| NMFI outpatient care | $3.00 | [3] |
| **Schistosomiasis** |  |  |
| Schistosomiasis cost of drugs | $0.25 | [14] |
| Schistosomiasis cost of individual MDA | $0.68 | [14]Average MDA cost per individual treated |
| Assumption of RDT multiplex cost when perspective is schistosomiasis only | $0.60 |  |
| **DALY- DISABILITY WEIGHT.  Disability weights for malaria as acute disease were scaled down to 2 weeks in a time horizon of 1 year (*2/52)** | | |
| **Malaria** |  |  |
| Malaria (severe) | 0.005 | [15] |
| Malaria (no complication) | 0.002 | [15] |
| **Non-malaria febrile illness (NMFI)** |  |  |
| NMFI (severe) | 0.005 | Assumption, same disability of malaria based on [7] |
| NMFI (no complications) | 0.002 | Assumption, same disability of malaria based on [7] |
| **Schistosomiasis** |  |  |
| Schistosomiasis (severe – schistosomiasis combined with anaemia) | 0.052 | [15] |
| Schistosomiasis moderate | 0.006 | [15] |
| **Death** | 1 |  |

**References**

1. *World Bank national accounts data, and OECD National Accounts data files*. 2019.

2. Ghai, R. R., Thurber, M. I., El Bakry, A., Chapman, C. A., Goldberg, T. L. (2016). Multi-method assessment of patients with febrile illness reveals over-diagnosis of malaria in rural Uganda. Malaria Journal, 15, 1-6.

3. Exum, N. G., Kibira, S. P., Ssenyonga, R., Nobili, J., Shannon, A. K., Ssempebwa, J. C., et al (2019). The prevalence of schistosomiasis in Uganda: A nationally representative population estimate to inform control programs and water and sanitation interventions. PLoS neglected tropical diseases, 13(8), e0007617.

4. Hawkes, M., Conroy, A. L., Opoka, R. O., Namasopo, S., Liles, W. C., John, C. C., et al. (2014). Use of a three-band HRP2/pLDH combination rapid diagnostic test increases diagnostic specificity for falciparum malaria in Ugandan children. Malaria journal, 13, 1-6.

5. Stothard, J. R., Kabatereine, N. B., Tukahebwa, E. M., Kazibwe, F., Rollinson, D., Mathieson, W., et al. (2006). Use of circulating cathodic antigen (CCA) dipsticks for detection of intestinal and urinary schistosomiasis. Acta tropica, 97(2), 219-228.6. Uzochukwu, B. S., Onwujekwe, E. O., Onoka, C. A., & Ughasoro, M. D. (2008). Rural-urban differences in maternal responses to childhood fever in South East Nigeria. PloS one, 3(3), e1788.

7. Shillcutt, S., Morel, C., Goodman, C., Coleman, P., Bell, D., Whitty, C. J., et al. (2008). Cost-effectiveness of malaria diagnostic methods in sub-Saharan Africa in an era of combination therapy. Bulletin of the World Health Organization, 86, 101-110.

8. Mnkugwe, R. H., Minzi, O. S., Kinung'hi, S. M., Kamuhabwa, A. A., Aklillu, E. (2020). Prevalence and correlates of intestinal schistosomiasis infection among school-aged children in North-Western Tanzania. PloS one, 15(2), e0228770.9. World Health Organization. (2013). Schistosomiasis: progress report 2001-2011, strategic plan 2012-2020. World Health Organization.

10. International Monetary Fund, I.F.S., *Official exchange rate (LCU per US$, period average)*. 2022.

11. Institute for Health Metrics and Evaluation (IHME). Health Service Provision in Uganda: Assessing Facility Capacity, Costs of Care, and Patient Perspectives. Seattle, WA: IHME, 201412.

12. Hansen, K. S., Ndyomugyenyi, R., Magnussen, P., Lal, S., Clarke, S. E. (2017). Cost-effectiveness analysis of malaria rapid diagnostic tests for appropriate treatment of malaria at the community level in Uganda. Health policy and planning, 32(5), 676-689.

13. Lubell, Y., Hopkins, H., Whitty, C. J., Staedke, S. G., Mills, A. (2008). An interactive model for the assessment of the economic costs and benefits of different rapid diagnostic tests for malaria. Malaria Journal, 7, 1-11.

14. Brooker, S., Kabatereine, N. B., Fleming, F., Devlin, N. (2008). Cost and cost-effectiveness of nationwide school-based helminth control in Uganda: intra-country variation and effects of scaling-up. Health policy and planning, 23(1), 24-35.

15. *Global Burden of Disease Study 2019 (GBD 2019) Disability Weights*. 2020, Institute for Health Metrics and Evaluation (IHME): Seattle, United States of America.
