# Supplementary material for: Cost-effectiveness of sentinel screening of endemic diseases alongside malaria diagnosis: A case study in schistosomiasis
Source: PLoS Negl Trop Dis. 2024 Jul 29;18(7):e0012339. doi: 10.1371/journal.pntd.0012339 (PMC11309411; doi:10.1371/journal.pntd.0012339)
Supplement: S1 Fig — (PDF) [file pntd.0012339.s002.pdf]

S1 Fig. Scenario analyses for the outcome cost/correct diagnoses

Reducing MDA coverage to 75%

Reducing WTP (one third)

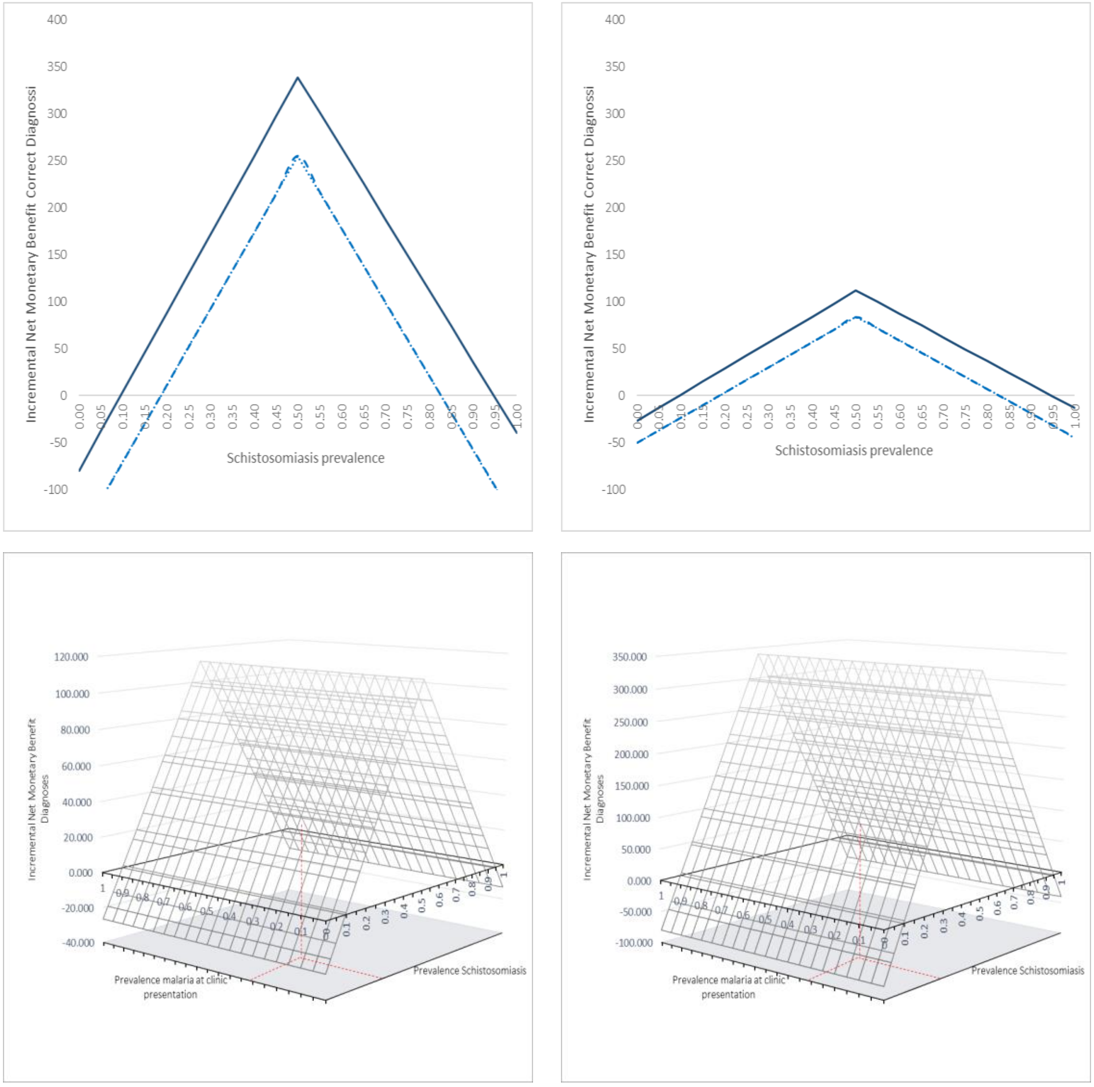

Incremental Net Monetary Benefit for every comparison across the two outcomes in the base case analysis. On scenario i) and ii) related to Correct diagnosis Schistosomiasis. Row represents NMB as function of schistosomiasis only (first row) or as function of both schistosomiasis and malaria (second row). Dashed lines represents relevant prevalence levels of schistosomiasis and malaria for the rural Ugandan context. Dark blue in the first raw represent the prototype, then dotted and dashed lines represent no cost savings and cost savings with current diagnostics respectively.
