# Supplementary material for: Cost-effectiveness of sentinel screening of endemic diseases alongside malaria diagnosis: A case study in schistosomiasis
Source: PLoS Negl Trop Dis. 2024 Jul 29;18(7):e0012339. doi: 10.1371/journal.pntd.0012339 (PMC11309411; doi:10.1371/journal.pntd.0012339)
Supplement: Attachment — Submitted filename: Response_to_Reviewers.docx [file pntd.0012339.s003.docx]

Ref: [PNTD-D-23-00349]

Manuscript Title: Cost-effectiveness of Screening of Endemic Diseases alongside malaria Rapid Diagnostic Testing: implications for mass drug administration policies.

Dear Editor,

My co-authors and I would like to thank you for considering our paper “Cost-effectiveness of Screening of Endemic Diseases alongside malaria Rapid Diagnostic Testing: implications for mass drug administration policies”.

The reviewers have provided excellent feedback for us on how to re-shape and present our ideas on this topic. Please find below, were applicable, our point-by-point response to the referees’ comments. Please note that since the aim, based on previous reviewers’ suggestions, has now changed, many comments are no longer applicable.

**METHODS**

-Are the objectives of the study clearly articulated with a clear testable hypothesis stated?

-Is the study design appropriate to address the stated objectives?

-Is the population clearly described and appropriate for the hypothesis being tested?

-Is the sample size sufficient to ensure adequate power to address the hypothesis being tested?

-Were correct statistical analysis used to support conclusions?

-Are there concerns about ethical or regulatory requirements being met?

**Reviewer #1**: It seems the primary question in this investigation is whether testing for schisto alongside malaria would be cost effective. I think this paper improves over the last version by taking malaria testing as a given rather than assessing whether it is cost effective. However, given this change, I am confused as to why so much of the model focuses on detailed malaria components, such as treatment for severe malaria in hospital settings, which seem to have no bearing on the research question. If the standard of care is taken as how fevers are tested and treated for malaria, then I would think the relevant model should focus on the additional costs and benefits provided by different means of testing and treating for schisto in routine clinical practice, and this detailed malaria decision tree could be removed entirely. Doing so would in my opinion greatly improve the clarity of this piece.

Throughout I remain very confused by the authors' use of the term "MDA". Mass drug administration is the practice of giving an entire population drugs via a campaign. It is not the same thing as presumptive treatment, in which a drug is given without a test result to a sick individual seeking care at a health facility. But as far as I can tell it is the latter that the authors have modeled. This is problematic both for clarity of the paper but also for its usefulness - MDA in the true sense is indeed the current standard intervention to reducing schisto prevalence, and thus a comparison of routine clinical testing and/or treatment vs a population MDA would be a policy relevant comparison. But from what I can tell, this investigation instead compares presumptive treatment of schisto for certain individuals (who these patients are, in what clinical setting, what symptoms they have, etc is never clarified) vs testing and treatment.

I question the assumptions around the hypothetical combined malaria-schisto test: it seems unlikely that it would be more accurate than any current test and yet cost about the same. As the authors note there has been considerable effort and resources invested in malaria tests, including making them more accurate. It seems likely that adding additional antigens to them would compromise accuracy, not improve it. The assumption that the test would necessarily be cheaper than two individual tests for a similar reason seems to me not to be a certainty - perhaps the method required to embed both antigens and ensure accuracy would require substantial additional cost.

This scenario thus seems overly speculative to me and produces the unsurprising finding that a perfect test would be superior to the tests we have in reality.

The outcome “incremental cost per additional right diagnosis” seems like a curious choice for comparison given that it cannot be relevant for the MDA scenario where diagnosis is not involved. I would have expected the modeleed outcome to be the cost per true infection appropriately treated, which would account for the cost of both diagnosis & treatment and allow for direct comparison between an MDA approach and a test and treat approach.

"This study examines costs from a payer perspective, making it relevant for public healthcare decision makers.” This seems incorrect in that, given the use of national GDP to set the threshold for cost-effectiveness, the paper seems to assume that patients will pay the cost of all diagnosis and treatment. That’s not how it works in most African countries where malaria diagnosis and treatment are free to patients (sometimes with a small flat user fee) at health facilities, and similarly the cost of MDA for neglected tropical diseases is covered by donors. I think this analysis would be more useful to decision makers if it considered who bears the costs.

**Authors’ response:**

Since the aim, based on previous reviewers’ suggestions, has now changed, many comments are no longer applicable. We understand that one of the main concerns was that the model “should focus on the additional costs and benefits provided by different means of testing and treating for schisto in routine clinical practice, and this detailed malaria decision tree could be removed entirely. Doing so would in my opinion greatly improve the clarity of this piece”. We feel that taking this approach would make or manuscript trivial as it will not embed the economy of scope, which is an essential element of our study.

**Reviewer #2**: I think this is mostly ok. I'm not sure about the NMB or EVCI equations. I think one-way SA on all parameters should be added.

**Authors’ response:** the NMB or EVCI equations are provided for completeness. Given the nature of our research question and the setting we have described, we feel that producing scenario analysis may be more informative than a one-way sensitivity analysis. We also feel that our scenario analysis covers aspects of what it could otherwise be called sensitivity analysis.

**Reviewer #3**: The authors have addressed previously raised concerns.

**RESULTS**

-Does the analysis presented match the analysis

-Are the results clearly and completely presented?

-Are the figures (Tables, Images) of sufficient quality for clarity?

**Reviewer #1**: Unfortunately the Figures are very low resolution and I cannot read them, especially the 3d panels. This makes it hard to follow the results.

**Authors’ response:** The issue with the resolution has now been resolved.

**Reviewer #2**: Tables and figures need further information so that they are clear as standalone items

**Authors’ response:** While these issues were not raised before, whenever possible, further info have been added to guide the interpretation of the Figures.

**Reviewer #3**: The authors have addressed previously raised concerns.

**CONCLUSIONS**

-Are the conclusions supported by the data presented?

-Are the limitations of analysis clearly described?

-Do the authors discuss how these data can be helpful to advance our understanding of the topic under study?

-Is public health relevance addressed?

**Reviewer #1**: (No Response)

**Reviewer #2**: Mostly, though I think they might be jumping too far in their conclusions if they find that the only parameters they explored have a large impact on results.

**Authors’ response:** The conclusions have now been reformulated to reflect the changes that have been made in the manuscript. Mostly, we now highlight the importance of sentinel screening with regard to testing patients compared with the status quo and with regards to surveillance.

**Reviewer #3**: The authors have addressed previously raised concerns.

**EDITORIAL AND DATA PRESENTATION /MODITICATIONS**

Use this section for editorial suggestions as well as relatively minor modifications of existing data that would enhance clarity. If the only modifications needed are minor and/or editorial, you may wish to recommend “Minor Revision” or “Accept”.

**Reviewer #1**: I would check your references - I did not review all but the refs for line 97 -- "The device prototype mentioned in the last objective is a novel point-of-care DNA assay device developed in collaboration with the Ministry of Health,

Uganda and tested in rural communities [11, 14]." -- appear to be more generic NTD reviews and do not seem relevant.

I think the caption for Fig 1 requires a citation of Phelps and Mushin (15) given it seems to be taken from that paper?

**Authors’ response:** We have now reviewed the references and the citation for Fig 1 has been added.

**Reviewer #2**: There's still quite a few typos

**Authors’ response:** The manuscript has now been checked for typos and spelling throughout.

**Reviewer #3**: The authors have addressed previously raised concerns. However, the article still requires a thorough check for typos and spelling. Tables need to be checked for consistency in case capitalisation etc. This is the only concern.

**Authors’ response:** The manuscript has now been checked for typos and spelling throughout**.**

**SUMMARY AND GENERAL COMMENTS**

Use this section to provide overall comments, discuss strengths/weaknesses of the study, novelty, significance, general execution and scholarship. You may also include additional comments for the author, including concerns about dual publication, research ethics, or publication ethics. If requesting major revision, please articulate the new experiments that are needed.

**Reviewer #1**: I think general clarification of methods and results would still be very helpful - having multiple scenarios and multiple outcomes, only some of which apply to each other, makes things challenging to follow (particular given the figures did not come through clearly). I'd recommend some simplification and streamlining of scenarios/outcomes.

**Authors’ response:**

Since the aim, based on previous reviewers’ suggestions, has now changed, many comments are no longer applicable. Whenever possible, we have now simplified scenarios and outcomes reported. These are: 1) Increase by two times the disability for schistosomiasis, 2) Decrease the coverage of mass drug administration MDA, 3) Decrease the willingness to pay (WTP).

**Reviewer #2**: Congrats on a much improved manuscript! Most of my comments are minor (bigger issues indicated with '*'

Abstract Aim – why not state which endemic disease here? (eg endemic disease using schitosomiasis as an example) line 57 – need to explain why you’d choose RDTs over gold-standard microscopy

paragraph line 109 feels disjointed. You could describe the burden of disease for any 2 diseases. Why these 2? How do they relate?

*Figure 1 – is the diagnostic accuracy combined for both tests? How do the different diseases relate?

Table 1 – Why are there two different malaria RDT sensitivities and specificities? Is first line treatment for malaria or schisto or both? (check all parameters are clear whether apply to malaria or schisto or both)

Fig 3 – would be helpful to explain what the comparators are

*Fig 4 – why are these scenarios and not sensitivity analyses? Why not do a one-way sensitivity analysis on the other parameters to see how those impact the results? I don’t think the first paragraph of your discussion is supportable if the only drivers you investigated are the key drivers. (How do you know this if you didn’t look at the others?) This becomes an issue again at line 431 paragraph.

*Could you add something in the discussion about what might happen if you want to screen for more than one disease in addition to malaria? How do decision makers decide which other disease to include? Does this limit the value of your analysis?

**Authors’ response:**

Since the aim, based on previous reviewers’ suggestions, has now changed, many comments are no longer applicable. With regards to bigger issues, clarifications for Figures 1 and 4 are now provided. However, as previously stated, given the nature of our research question and the setting we have described, we feel that producing scenario analysis may be more informative than a one-way sensitivity analysis. Further in the discussion section, but in general throughout the manuscript, we describe scenarios where malaria is tested alongside schistosomiasis.

Potentially, decision makers may decide to include other diseases in they model; while the flexibility of our model allows so, suggesting what disease should be included is beyond the scope of this manuscript.

**Reviewer #3**: The authors have addressed previously raised concerns.
