# Supplementary material for: Cost-effectiveness of sentinel screening of endemic diseases alongside malaria diagnosis: A case study in schistosomiasis
Source: PLoS Negl Trop Dis. 2024 Jul 29;18(7):e0012339. doi: 10.1371/journal.pntd.0012339 (PMC11309411; doi:10.1371/journal.pntd.0012339)
Supplement: Attachment — Submitted filename: Comments on cost-effectiveness of screening of Endemic Diseases alongside malaria diagnosis.docx [file pntd.0012339.s004.docx]

**Cost-effectiveness of screening of Endemic Diseases alongside malaria diagnosis**

*Comments*

1. The paper brings out a pertinent issue on combination of screening tests which in my view would greatly be cost saving and more acceptable to population and health authorities. I have worked in an environment where parallel programs for each disease created fatigue to all parties concerned! It would also strengthen the current advances in science. It could be perfected over time and that has been the case with the evolution of malaria RDT.
2. I propose consideration be made to change heading for example “cost-effectiveness of screening of endemic diseases alongside malaria diagnosis; a case of schistosomiasis”. In my understanding, it is schistosomiasis under review though a wider perspective of other endemic diseases is to limited extent mentioned (e.g helminths). Of essence, the gist of the paper is malaria and schistosomiasis. This could simultaneously change the main objective of the research.
3. Iam cognizant that the paper examines costs from the payer perspective who could be government, health insurance body or the patient in case of out-pocket payment. In my view this could be okay as long as all the costs are captured.
4. The decision trees do not reflect the additional benefit of screening for schistosomiasis and any other NTDs. The trees more less represent outcome of decisions around malaria illness. Iam inclined to question their relevancy to the study.
