# Supplementary material for: Cost-effectiveness of sentinel screening of endemic diseases alongside malaria diagnosis: A case study in schistosomiasis
Source: PLoS Negl Trop Dis. 2024 Jul 29;18(7):e0012339. doi: 10.1371/journal.pntd.0012339 (PMC11309411; doi:10.1371/journal.pntd.0012339)
Supplement: Attachment — Submitted filename: Response_to_Reviewers.docx [file pntd.0012339.s005.docx]

May 28, 2024

Syamal Roy
Academic Editor

*PLOS Neglected Tropical Diseases*

Manuscript ID:	PNTD-D-23-00349

Manuscript title:	Cost-effectiveness of sentinel screening of endemic diseases alongside malaria diagnosis: a case study in schistosomiasis

Dear Professor Roy:

I am writing to you in relation to the manuscript titled ‘Cost-effectiveness of sentinel screening of endemic diseases alongside malaria diagnosis: a case study of schistosomiasis’ which was submitted initially to PLOS NTD by Francesco Manca and co-authors for consideration for publication. Following its initial review, we were asked to further amend the manuscript in light of the reviewers’ comments. We are pleased that reviewers appreciated our manuscript, stating that it was *well written* and addressing *pertinent issues.* We have carefully reviewed the reviewers comments and have revised the manuscript accordingly. Our responses are detailed below in a point-by-point manner. We provide two versions of the manuscript, one with tracked changes and the other one ‘clean’.

I kindly ask to add myself (Francesco Manca) as additional corresponding author, as I was on long-term absence from work and co-authors helped in liaising with the journal and the submission process, but I am now available and willing to be a corresponding author for this manuscript.

We very much hope that the revised manuscript is now acceptable for publication. We look forward to hearing from you in due course.

Sincerely,

Francesco Manca

Health Economics and Health Technology Assessment

School of Health and Wellbeing

Clarice Pears Building 90 Byres Road

University of Glasgow

Glasgow G12 8TB Email: franesco.manca@glasgow.ac.uk

Editorial summary

Thank you very much for submitting your manuscript "Cost-effectiveness of Screening of Endemic Diseases alongside malaria Rapid Diagnostic Testing: Implications for mass drug administration policies" for consideration at PLOS Neglected Tropical Diseases. As with all papers reviewed by the journal, your manuscript was reviewed by members of the editorial board and by several independent reviewers. The reviewers appreciated the attention to an important topic. Based on the reviews, we are likely to accept this manuscript for publication, providing that you modify the manuscript according to the review recommendations.

Overall the comments of the reviews are favourable. The authors are suggested to respond to the concerns raised by reviewer #4 and minor concerns by other reviewers

**Answer:** Thank you for your time reviewing our study and reviewers’ comments. We have provided a revised version of the manuscript considering all reviewers’ points. Please see our answers below.

Reviewer's Responses to Questions

**Methods**

Reviewer #1: 1.	The paper brings out a pertinent issue on combination of screening tests which in my view would greatly be cost saving and more acceptable to population and health authorities. I have worked in an environment where parallel programs for each disease created fatigue to all parties concerned! It would also strengthen the current advances in science. It could be perfected over time and that has been the case with the evolution of malaria RDT.

**Author response:** Many thanks for your valuable feedback. We have incorporated all your comments, detailed below, in our revised version of the manuscript. We can see how much these comments have improved our paper’s message and transparency.

2. I propose consideration be made to change heading for example “cost-effectiveness of screening of endemic diseases alongside malaria diagnosis; a case of schistosomiasis”. In my understanding, it is schistosomiasis under review though a wider perspective of other endemic diseases is to limited extent mentioned (e.g helminths). Of essence, the gist of the paper is malaria and schistosomiasis. This could simultaneously change the main objective of the research

**Author response:** Many thanks for your suggestion. The title has been changed accordingly. We have also included the word ‘sentinel’ in the title to reflect your sentiment.

Reviewer #2: -Are the objectives of the study clearly articulated with a clear testable hypothesis stated? Yes -Is the study design appropriate to address the stated objectives? Yes -Is the population clearly described and appropriate for the hypothesis being tested? Yes -Is the sample size sufficient to ensure adequate power to address the hypothesis being tested? Yes -Were correct statistical analysis used to support conclusions? Yes -Are there concerns about ethical or regulatory requirements being met? NO

**Author response:** Many thanks for your feedback.

Reviewer #3: Yes, all reviewer's comments are addressed

Reviewer #4: Review of manuscript titled “Cost-effectiveness of Screening of Endemic Diseases alongside malaria Rapid Diagnostic Testing: Implications for mass drug administration policies”

The study aimed to present an economic model demonstrating the potential added value of "sentinel” screening for schistosomiasis, in addition to malaria diagnosis, for febrile patients at healthcare facilities.

The authors have explored the cost-effectiveness of sentinel screening by modifying the existing healthcare protocol, considering three different comparators and three additional scenarios to generalize the outcomes for other settings and diseases.

While I appreciate the concept behind the study involving sentinel screening, the execution and presentation are not systematically done.

Mainly, almost all the outcomes are context and variable dependent, thereby limiting the study’s scope and reliability.

The quality of provided images, particularly in Figure 1 and Figure 2, is suboptimal and hard to follow. Figure 3 and Figure 4 need better representation, and legends, especially for Figure 2, are lacking.

Numerous typos, poorly formulated sentences were noted throughout the manuscript. In my opinion, the manuscript requires significant reformatting in terms of figure presentation, results, and discussion.

Considering these concerns, I have decided to reject the manuscript in its current form.

**Author response:** Many thanks for your observations and comments.

We understand the concern that most of the outcomes are context and variable dependent and we generally appreciate the critique on the sensitivity of our results. Indeed, our original intention was to design a flexible model, adaptable to different contexts and diseases. We accept that this was not initially sufficiently clear. We have therefore rephrased our study’s objectives to make this explicit.

Additionally, we now include the following improvements:

The literature reports economies of scale for MDA as an extra cost-effectiveness driver. Whilst previously, this was only briefly considered in the discussion, a more detailed scenario analysis has now been included.

Regarding the sensitivity of the model to key variables, we have stressed this in our limitations. In order to include extreme cases referring to different diseases (with, for example, twice the disability weighting) or willingness to pay (for a significantly reduced amount), we have ‘extended’ our model, showing the effect of different results.

Regarding Schistosomiasis, we also report that policy decisions on which strategy (MDA vs testing) and on which test to use (based on cost and accuracy) were already found to be highly context specific (P. T. Hoekstra, (2021); Hoefle-Bénard, J., & Salloch, S. (2024)). Our model re-emphasises this and, with the additional sensitivity analyses conducted, we highlight the drivers of cost effectiveness in different circumstances. In Uganda, costs can vary significantly within the country across different rural areas (Brooker, S., et al. 2008) and as such ‘blanket polices’ are likely to be less effective when considered in different contexts.

We also acknowledge that our sensitivity analyses are not defined to be exhaustive but we believe they may help highlight differences in results when assumptions driving cost-effectiveness change.

We have extensively reframed our ‘limitation section’ to include the aforementioned points.

Images have been up-graded and the quality improved in line with imagine file sizes, as per journal requirements

The manuscript has been carefully reviewed, and typographical errors, grammar and syntax have all been improved.

**Results**

Reviewer #1: The decision trees do not reflect the additional benefit of screening for schistosomiasis and any other NTDs. The trees more less represent outcome of decisions around malaria illness. I am inclined to question their relevancy to the study.

**Author response:**

Thank you for your comment and apologies this was not initially clearer.

Since the main objective of the paper is to compare the cost-effectiveness of opportunistic screenings of potentially multiple diseases (schistosomiasis is simply a case study) along a malaria diagnosis, we preferred to keep the schistosomiasis tree generic in order to facilitate greater model flexibility (i.e. to be adaptable to other diseases). The model is focused on patients having a malaria test at clinics regardless of the Neglected Tropical Disease (NTD) test they need; therefore, we developed the malaria part of the tree (decision tree A) in greater depth since this represents the common component of the tree.

Regarding our overall decision tree model:

Overall, we assessed three comparisons in the manuscript.

In the first two comparisons we analyse only changes in the ‘schistosomiasis device’ regarding its sensitivity and fixed costs (keeping the malaria side of the device constant). In the third comparison we analyse changes in both the schistosomiasis and malaria sides of the devices. This was originally to offer three different scenarios where there is a different extent of economies of scope and device accuracy.

For the first two comparisons the malaria decision tree is redundant and ‘cancels out’ into the evaluation. In the third comparison the malaria decision tree comes into play as we compare a more accurate (sensitivity and specificity increased) malaria device together with a more accurate schistosomiasis device.

To clarify these aspects as being part of the goal of the paper, we have now written this into the main text as follows: *For comparator 1 and 2, the diagnosis for malaria was the same for both strategies and this would cancel out in an incremental evaluation. Therefore, the cost effectiveness of the model was dependent only on schistosomiasis prevalence and the relevant model is only the Schistosomiasis part of the tree (Figure 2 B). In contrast, for comparison 3, with improved sensitivity and specificity of the prototype for both malaria and schistosomiasis, the overall diagnoses can change, and the NMB of the device was a function of both malaria and schistosomiasis prevalence.*

Reviewer #2: -Does the analysis presented match the analysis plan? Yes -Are the results clearly and completely presented? yes -Are the figures (Tables, Images) of sufficient quality for clarity? Some are not of good clarity in the downloaded version. Mentioned in the detailed review.

**Author response:** Images have been changed as described previously and in-line with journal requirements

Reviewer #3: Yes

Reviewer #4: The analysis does not match the analysis plan

The results are not clear

Figures are also not easy to follow

**Author response:** We have inserted subheadings to better describe our approach and facilitate greater clarity on the presentation of results. We have also changed some of the images to facilitate this

--------------------

**Conclusions**

Reviewer #1: I am cognizant that the paper examines costs from the payer perspective who could be government, health insurance body or the patient in case of out-pocket payment. In my view this could be okay as long as all the costs are captured.

**Author response:** Thank you. We used several different costing sources to include costs for the payer perspective. All costs were inflated, converted and extensively reported in the supplementary materials.

Reviewer #2: -Are the conclusions supported by the data presented? yes -Are the limitations of analysis clearly described? partly -Do the authors discuss how these data can be helpful to advance our understanding of the topic under study? Yes -Is public health relevance addressed? Yes

**Author response**: Our discussion on limitations has been extended, as described above, and as suggested by other reviewers.

Reviewer #3: Yes

Reviewer #4: Conclusions are not completely supported by the data

Limitations are not highlighted

The authors do discuss how these data can help understanding the topic

Public health relevance is discussed

**Author response:** Our conclusions and limitations have now been amended accordingly and we hope, are more reflective of our model. Specifically, regarding limitations, we have now highlighted the relevance of costs, the fact that we did not model parasite transmission dynamics related to MDA and logistic specificities on MDA delivery. However, all these factors are now fully discussed and commented on in discussion under ‘limitations’.

e.g. see: *We acknowledge that the model we presented is highly sensitive to specific parameters (e.g., MDA coverage as well as diagnostic accuracy and costs- see differences between comparator 1 and 2). […] Parasite transmission dynamics were not modelled, but we believe that they would decrease the effectiveness of intervention; but remain not crucial for a model with a 1-year time horizon.*

The overall aim of the study was to provide a general model adaptable to different contexts, therefore all our sensitivity analyses are not exhaustive of all the possible scenarios. We also now highlight this as a limitation (as well as a strength). This is explicated in the manuscript in the limitations section:

*“[…]Finally, we are aware that our sensitivity analyses are not exhaustive, but we believe they may point to the direction of the difference in results when some assumptions driving cost-effectiveness change.”*

--------------------

<b>Editorial and Data Presentation Modifications?</b></br><br/> Use this section for editorial suggestions as well as relatively minor modifications of existing data that would enhance clarity. If the only modifications needed are minor and/or editorial, you may wish to recommend “Minor Revision” or “Accept”.

Reviewer #1: Nil

Reviewer #2: Have mentioned some points which if discussed could add value to the paper , Highlighted in the detailed review.

Reviewer #3: Minor changes in figures resolution.

Reviewer #4: (No Response)

--------------------

**Summary and General Comments**

Reviewer #1: Nil

Reviewer #2: The manuscript PNTD-D-23-00349 entitled “Cost-effectiveness of Screening of Endemic Diseases alongside malaria Rapid Diagnostic Testing: Implications for mass drug administration policies” is overall a **well written** paper. The article focuses on the additional value, of sentinel screening for schistosomiasis during routine malaria diagnosis of patients presenting to malaria clinics with fever.

The study presents the results from comparative analytical models for the same and suggests the main points identifying the drivers enabling cost effectiveness of the approach which would allow policy deliberations regarding the cost effectiveness of the approach in different endemic regions where both schistosomiasis and malaria are endemic. The present study focuses on sub–Saharan Africa where both the named diseases are co-endemic, and there remains significant morbidity and mortality especially in resource challenged settings.

The analytical model in the paper was based on the approach of Phelps and Mushin, the highlights of which have been mentioned. The model was used to evaluate a healthcare protocol, with a reference population of school going children, as they are considered to be the most affected by these two diseases in this region and additionally most exposed to mass drug administration for schistosomiasis.

The model compared two healthcare protocols for individuals with fever 1) the prevalent health care using diagnostic test based treatment for malaria and MDA for schistosomiasis.

2) a protocol using a diagnostic test for malaria and a test for schistosomisasis.

The model was used to assess cost effectiveness of such a health protocol in the Ugandan setting via three analyses, a) Comparison of a new protocol testing for malaria and sentinel screening for schistosomiasis with currently available devices.

b) Comparison of a new protocol testing for malaria and sentinel screening for schistosomiasis with both diagnostics in one device only.

c) Comparison of a new protocol for malaria and a sentinel screening for schistosomiasis with a new device showing higher accuracy and both diagnostics in the same device.

The analyses suggests that the accuracy and diagnostic device (as expected) along with factors like the MDA coverage and other context specific parameters should determine the prevalence cut offs for deciding diagnostic RDT or MDA based policies.

While an overall **well written** article the fact remains that all analyses performed will remain inextricably linked to the sensitivity, specificity and usage conditions including expertise of the operators and other environmental factors. Thus it may be difficult to compare two studies from different locations for purposes of deciding policy. Also as suggested, to extend this approach of sentinel screening to other NDTs would first require the availability of suitable devices with multi pathogen detection capability. While this is certainly desirable, it will not be easy to achieve given our incomplete information about many of these pathogens. In fact even in the case of malaria there is a huge scope for more advanced, affordable specific and sensitive devices. What attracted me about the use of sentinel screening-based approaches is the avoidance of unnecessary use of drugs many of which could have possible side effects.

It would be nice if the authors could add a few comments based on the implications mentioned in the previous paragraph.

Minor point

Some of the figures, such as Fig.2 appeared to be extremely fuzzy in the version downloaded. If this could be kindly checked.

**Author response:** Thank you for these observations. We have addressed these considerations in the discussion as follows:

**“***For ease of understanding, we classified the drivers determining the prevalence cut offs into four categories:*

*1) Device components (accuracy -sensitivity and specificity- and economies of scope - higher cost savings from detecting two diseases simultaneously increases chances of the device being cost effective)*

*2) MDA program features (cost of treatment, economies of scale- reduction of single treatment costs when increased the number of individuals treated- and MDA coverage -lower percentages make ‘treat all’ MDA strategy less effective than high accuracy testing in high prevalence setting-)*

*3) Environmental components (local capacities and expertise, disease prevalence). We first go through them below and then we discuss them in detail.*

*4)Perspective -analysing the healthcare protocol overall, considering health gains coming from both diseases increase the likelihood of a new protocol including multiple detections to be cost-effective-.*”

**[…]**

**“***Lastly, the model could also embed the additional value of avoiding treatment side effects for individuals tested negative compared to MDA. This could be done in the model (Figure 2) by adding disability weights in all treated individuals. This would increase the EVCI of the test and can be particularly useful for treatment with severe side effects. However, as side effects from praziquantel, whilst common, are usually mild and temporary, we do not model this in our case study referring to schistosomiasis.*“

As mentioned to reviewer 4, we agree that our model is sensitive to assumptions and contexts. We included this into the discussion section. We believe that changing some key parameters driving the cost-effectiveness in a one-way sensitivity analysis and describing new scenarios, we provided hints on how results in different contexts may vary. We broadly discussed this and in the limitations section we stated the text below which we believe can synthetise these concepts:

*“[…]Finally, we are aware that our sensitivity analyses are not exhaustive, but we believe they may point to the direction of the difference in results when some assumptions driving cost-effectiveness change.”*

Reviewer #3: Overall a good study on the feasibility of screening of endemic diseases alongside malaria Rapid Diagnostic Testing

**Author response:** We thank reviewer for this comment

Reviewer #4: All together the data presented in this manuscript does not support the conclusions drawn. The study design is not fully scientifically justified.

**Author response:** We thank reviewer for this comment. We have now revised the conclusions, as described above and in light of the fully revised paper. In particular our conclusions now focus on our main results and sensitivity, see below:

*At average levels of prevalence of schistosomiasis in Uganda, a sentinel screening with current diagnostics and costs is likely to be not cost-effective. However, when we considered suboptimal levels of MDA coverage (as likely to be in the country), for high levels of schistosomiasis prevalence, economies of scope can make sentinel screenings using device with current accuracy cost-effective. Overall, sentinel screening for neglected tropical diseases such as schistosomiasis can be a relevant cost-effective alternative to MDA, however, decisions are context and device specific. By highlighting the importance of the epidemiological setting in determining the best strategy, the model results show how blanket thresholds recommended by international guidelines may not always be the most cost-effective strategy for endemic diseases.*
